# Environmental Impact of Burning Composite Materials Used in Aircraft Construction on the Air

**DOI:** 10.3390/ijerph16204008

**Published:** 2019-10-19

**Authors:** Iveta Vajdová, Edina Jenčová, Stanislav Szabo, Lucia Melníková, Jozef Galanda, Malgorzata Dobrowolska, Jindřich Ploch

**Affiliations:** 1 Department of Air Transport Management, Faculty of Aeronautics, Technical University of Kosice, Rampová 7, 04121 Kosice, Slovakia; iveta.vajdova@tuke.sk (I.V.); lucia.melnikova@tuke.sk (L.M.); jozef.galanda@tuke.sk (J.G.); 2 International Center for Interdisciplinary Research, Silesian University of Technology, 44-100 Gliwice, Poland; malgorzata.dobrowolska@polsl.pl; 3 College of International and Public Relations Prague, o.p.s., U Santošky 17, 150 00 Prague 5, Czech Republic; ploch@vsmvv.cz

**Keywords:** ecology, composite materials, air pollution, flammability, aircraft construction material, air incidents and emergency situations, safety

## Abstract

The negative impact of air incidents and emergency situations results from the leakage of liquids into the soil and water and the leakage of flue gases and combustion products of aircraft structural materials into the air during fires. This article deals with air pollution caused by the combustion of composite materials commonly used in general aviation. Samples of composite materials of aircraft registered in the Czech Republic were selected. These samples of composite materials were tested for flammability, according to ISO 5660-1:2002 Reaction to fire tests—Heat Release, smoke production and mass loss rate (ISO—International Organization for Standardization). Total smoke release and total oxygen consumed were assessed in this study, both of which have a significant impact on air quality in the case of an air incident. Based on the results of the research, differences resulting from the diversity of the structures of the tested composite materials were found. The most hazardous composite material was evaluated from the point of view of its impact on air quality during combustion.

## 1. Introduction

Air transport is one of the fastest-growing sectors, mainly because of the fluctuation that results from economic development and employment growth. Nowadays, people travel enormous distances in a short time, further promoting international travel and hence the demand for air travel. According to statistics, the number of flights increased by 80% between 1990 and 2014, and by 2035, it is estimated to increase by a further 45% [1]. Aviation, like every mode of transport, is responsible for a significant negative impact on the environment. Risks that accompany this mode of transport are, in particular, air pollution, excessive aircraft noise at and around airports, soil and water pollution, waste production with a high proportion of hazardous waste, and more [2]. In recent years, the environmental impact of aviation has increased as a result of the growth in air traffic. However, aviation companies are struggling with the negative environmental impacts of this industry, the technological innovations introduced, the renewal of the aircraft fleet, the increasing efficiency of Air Traffic Management and the economic downturn in 2008—emissions and noise from air traffic in 2014 were at the same levels as in 2005 [1].

However, air transport is not the only mode that adversely affects the environment, and many studies address the issue of reducing the negative impact of particular modes of transport. The study of Coffin, A.W. [3] deals with the destruction of ecosystems during the construction of roads and transport infrastructure, which affects the dynamics of ecosystem functioning and has direct effects on ecosystem components, including their species composition. The research of Macias, A. and Gadziński, J. [4] assesses the quality of the natural environment and the negative impact of road transport and identifies the conflict between the communication routes and the natural environment in the Poznań conurbation. The author used the model LATINO modified and adapted to local Polish conditions.

The greening of road and rail container transport is part of the study by Sun, Y. et al. [5], in which the authors explore the problem of routing containers at the operational level in a network of multimodal road and rail services. In order to reduce the negative impact of multimodal transport on the environment, the authors examined two kinds of optimization: the emission charging method and bi-objective optimization to optimize the CO_2_ emissions in the routing. Based on the results of the research, the bi-objective optimization method appears to be more effective for reducing CO_2_ in the present case. The issue of raising environmental standards is part of the research of Demir, E. et al. [6], in which the authors focus on intermodal transport. According to the authors, intermodal transport networks offer flexible, robust and environmentally sound alternatives to the transport of large volumes of goods over long distances. In this paper, the authors address the Green Intermodal Service Network Design Problem with Travel Time Uncertainty (GISND-TTU). Their stochastic approach allows the creation of robust transport plans according to different targets, which can be used in real conditions.

As already mentioned, air transport is a major contributor to the negative environmental impact but is not the only one. Global environmental change is a general problem. The author Forsyth, P. [7] combines the achievement of environmental sustainability in air transport with the financial sustainability of air transport. According to the author, it is possible to achieve the environmental and financial sustainability of aviation if effective policies are adopted, but this will have an impact on the use of non-renewable resources.

Authors Polishchuk, V. et al. [8] have developed a fuzzy model to assess the environmental risk of launching projects in the air transport sector at the expansion stage. The model should provide a tool to support decision making in the allocation of investment funds and the financing of environmental start-up projects.

Ecology is currently a sharply monitored trend in various areas of economy and transport. Air transport has many advantages (such as the speed of transport and safety) over other modes of transport. The environmental impact of aviation is not negligible, despite the fact that many important aviation organizations are constantly assessing the level of this impact and are taking appropriate action accordingly. Air transport, among other things, has an impact on air quality, especially in areas where airports are situated because most emissions from combustion engines are produced during the take-off and landing phases. Aircraft manufacturers are developing new types of drives and incorporating new types of materials into aircraft production to reduce the environmental impact of aviation. Despite the adoption of measures to improve aviation safety, air accidents are constantly occurring, which also has a negative impact on the environment. Take-off and landing are the most critical phases of flight in terms of safety and environmental impact [9]. The issue of the environmental impact of aviation is the focus of many studies. Air is most affected by the combustion of hydrocarbon fuels in vehicle combustion engines, where the formation of toxic and carcinogenic substances is involved as well as substances involved in the global warming of the Earth’s atmosphere (CO_2_, N_2_O, CH_4_). As the volume of air traffic continues to increase, addressing its environmental impact is more important [10]. Studies dealing with the impact of air traffic on air quality include, for example, Zhu et al. [11], which deals with air pollution during the take-off and landing phases when most emissions from exhaust gases are produced. The authors address the optimization of fuel loss and the reduction of the greenhouse effect by reducing additional aircraft load, using inventive air transfer control methods, and identifying the best performing aviation systems for all conditions. The study’s conclusions offer answers to the question of the relationship between aircraft descent and fuel consumption by searching for fuel-efficient descent, designing a new descent algorithm, and providing a more efficient aircraft descent design for pilots in addition to the Aircraft Crew Operations Manual (FCOM). Cao et al. [12] discussed a bottom-up CO_2_ emission analysis for the period 2006–2016 and an analysis of the CO_2_ emission impact factors of the Pearl River by the Logarithmic Mean Divisa Index (LMDI) decomposition method. The top-down analysis has been addressed by Liu et al. [13], which offer comprehensive aviation emissions in China for the period 1980–2015. Research results show that emissions in central and eastern China are much higher than in northeastern and western China. Based on the results, the authors offer a prediction by 2050, where they predict emission reductions using new technologies, but this will be offset by increased traffic volumes. In a study by Matveev et al. [14], the authors presented an algorithm for simulating CO and CO_2_ emissions in a model combustion chamber under various initial conditions. The results were compared with the results of proven experimental data, thereby verifying the impact of gas turbine engines (GTEs) on the air.

However, air transport does not only affect the environment directly by carrying out the service of transportation itself but also indirectly, for example, by air, soil, and water pollution in the case of incidents and emergency situations. Despite the fact that aviation safety is one of its fundamental priorities, air incidents are an integral part, as is the case with other transport sectors [15]. Aviation safety is dealt with by annual safety reports from international aviation organizations such as ICAO (International Civil Aviation Organization), IATA (International Air Transport Association), FAA (Federal Aviation Administration), and many others. Despite the increasing efforts of these components, incidents and emergency situations are occurring, and their environmental impact is not negligible. An analysis of airline incidents is discussed in the study of Li [16], where the author studied the trend of the variability of long-term historic accident data and their victims by means of a time series analysis, a Mann–Kendall trend analysis and by predicting possible changes in future air accidents and provided research on aviation safety and security. Air transport and, therefore, aviation technology is constantly evolving, and new materials are available that are well suited to aviation applications for a variety of reasons. Such materials are also composite materials that have become an integral part of aircraft designs. Despite the operational advantages of these materials, the composite materials in the aircraft structure also have disadvantages which result from the fact that the weakening of the inner structure of this structural material cannot be easily determined with the composite sandwich structure. Individual technological aspects of composite materials used in aerospace design and structure are reported by Dutton et al. [17]. The authors discuss important technological differences in composites and metals, addressing the differences in the effects on manufacturing processes, construction procedures and material performance during operation, in particular, the causes and nature of damage that may be caused during operation. In the context of airline incidents and used aircraft construction materials, heat performance in aircraft fire during an aircraft incident is an important issue.

As we deal with the flammability of composite materials in the presented research, we investigated the research in this field to examine the flammability tests of composites. Fires involving composite materials can release toxic fumes and microparticles into the air, causing health risks. The thermal properties of laminate composites are discussed in the research of Dodds et al. [18]. The authors investigated the thermal response of fiberglass-reinforced composite laminates, where composites have been shown to have excellent fire resistance.

Unlike the above, Lyon et al. [19] dealt with the fire resistance of carbon fiber composites made of potassium aluminosilicate (Geopolymer). The results were compared with organic matrix composites used for transport, military, and infrastructure applications. According to research results, carbon fiber materials with a geopolymer matrix retained 67% of their original flexural strength. Other investigations that address the flammability of composite materials include Mouritz and Gibson [20], which deals with the fire performance of polymer composite materials, Brown et al. [21] on the evaluation of the flammability of composite materials by cone calorimeters, Kozlowski and Wladyka-Przybylak [22] on the flammability and fire resistance of natural fiber-reinforced composites, and an overview of the flammability of natural composites and strategies for flame retardation by Chapple and Anandjiwala [23].

The answer to the question of the thermal properties of composite materials and the improvement of their fire properties is given, for example, by Zhou et al. [24], in which the authors discuss the development of a novel structure of Bismaleimide/diallyl bisphenol A (BD) commonly used in aviation. The new proposed and synthesized hierarchical structure of MoS_2_ and TiO_2_ improves the mechanical and fire safety of BD. Their results provide a new strategy for designing a modern BD composite material. Another study on the development of a composite material with better fire properties is presented by George et al. [25], in which the authors address a new nanocomposite for space applications.

Numerous studies have been conducted on the flammability of composite materials, but none of these studies deals with the environmental impact of composite materials in the event of a material fire in an air incident/accident. In order to obtain the information in question, this study focused on composite materials commonly encountered in structures of general aviation aircraft registered in the Czech Republic.

The motivation for dealing with the negative impact of air accidents on the environment results primarily from the lack of information on the issue at home and abroad. Aviation emergencies are part of air travel and, although globally their rates have declined in terms of flight numbers since 2014 (3.0 in 2014; 2.6 in 2018), the number of incidents and accidents has not declined significantly over the years [26]. Assuming the expected increase in air traffic in the future, the rate of accidents is likely to decrease in relation to their numbers, but we cannot assume that the number of such accidents will have a decreasing tendency. International organizations, however, pursue agendas solely on aviation incidents in the category of planned commercial flights of airliners whose take-off and landing mass ranges from 5700 kg maximum take-off mass (MTOM). Based on the statistical investigation carried out in the Czech Republic in the period 2006–2017, we found that air accidents and incidents of aircraft up to 2000 kg MTOM significantly exceed the accidents of transport aircraft in the given territory. As a result of this statistical investigation carried out in the framework of the previous authors’ research, it was evaluated that, between 2006 and 2017, there were 233 aircraft incidents of aircraft up to 2000 kg MTOM registered in the Czech Republic that in some way (leaking fuel, fire, etc.) disturbed the ecological biosphere at the event location. In 2017, there were 23 aviation incidents, and it should be noted that aviation incidents in that category of aircraft do not have a downward trend. The category of aircraft up to 2000 kg MTOM is given relatively little attention, although this category of aircraft records statistics of more aviation incidents (based on findings in the Czech Republic).

The present article provides information on research dealing with the greening of aviation. The main idea of this article is to discuss the environmental impact of accidents involving aircraft up to 2000 kg MTOM and in this study, we mainly focus on air pollution resulting from the burning of composite materials that are part of the construction of the aircraft category in question. After identifying research on the solved issue, attention is paid to the identification and description of the composition of materials that have been tested by the cone calorimeter method and that occur in aircraft structures of the aircraft category in question. The next section describes the method used. As part of the results, the dominant material for each aircraft type of the aircraft category in question is evaluated. The results of the flammability test by the cone calorimeter method provide information on the values of the monitored parameters, and conclusions are drawn on this basis.

## 2. Materials and Methods

**Figure 1:**
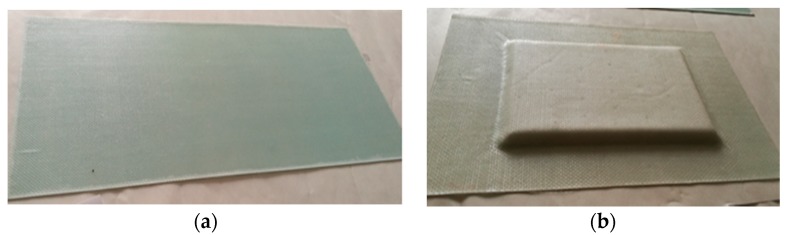
Example of composite material without core (**a**) and with core (**b**).

In connection with the solution of the project “Simulation of Intervention in Air Accidents”, an analysis of structural and interior materials of aircraft up to 2000 kg MTOM (maximum take-off mass) registered in the Czech Republic was performed. These materials were obtained from aircraft manufacturers or other sources, and samples were tested for flammability. Out of all the collected construction materials, which were tested in two sets, 7 samples of structural composite materials of different compositions were selected for the purpose of the research. All of the composite materials examined were sandwich core and non-core (core-free) structures, with the particular composition of the individual samples and the view of the core and core-free composite material being shown in Figure 1.

Material flammability was assessed according to ISO 5660-1 using the cone calorimeter method [27]. We focused mainly on time-dependent variables of fire, which affect the air quality at the place of combustion. In this research, the aim was not to provide a new method of testing the materials in question but to point out the negative impact of the combustion of composite materials used in aircraft structures, as these parameters and testing for that aim are not currently performed on structural materials. Samples were matched by their parameters and composition to the test requirements of the appropriate standard. The cone calorimeter can be used in tests of both homogeneous and heterogeneous samples that meet the standard prescribed parameters (including composite materials). The work of the cone calorimeter is based on the principle of oxygen consumption, which is based on the direct proportion between the amount of heat released and the amount of oxygen required for combustion. It has been empirically found that most fuels consume 13.1 × 10^3^ kJ/kg oxygen. The cone calorimeter measures the exact oxygen concentration in the exhaust port, and the volumetric airflow indicates the rate of oxygen consumption from which the heat released can be calculated. In the calorimeter, the rate of heat release is given by the relationship [28,29]:

(1) $$\dot{q}=\left(13.1\times 10^{3}\right)\times 10.1C\frac{\left(0.2095-X_{O_{2}}\right)}{\left(1.105-1.5X_{O_{2}}\right)}$$

where:

$\dot{q}$ = the rate of heat release (kW);

C = the orifice plate coefficient (kg12m12K12);

$X_{O_{2}}$ = the measured mole fraction of O_2_ in the exhaust air.

The cone calorimeter is used to assess the rate of heat evolution during sample ignition, with the test method being determined for small representative samples (ISO 5660-1:2002). The standard sample size is 100 × 100 mm^2^. The maximum allowed sample thickness is 50 mm. The sample is wrapped in aluminum foil so that only the top of the sample is exposed to heat and fire radiation with a cone calorimeter. Before placing the lighter over the sample surface and starting the test, the baseline data is collected for 60 s. The experimental data is then measured from this baseline [29]. The cone calorimeter measurement results can be used for various purposes [30].

When measuring the flammability of selected materials, several parameters such as the Effective Calorific Value (MJ/kg), Maximum Heat Release Rate (kW/m^2^), Oxygen Consumed (g), Smoke Release Rate (m^2^/m^2^), MARHE (Maximum Average Rate of Heat Emission) (kW/m^2^) and Combustion Residue (g) were evaluated [31]. Samples were exposed to heat (fire) radiation from both sides, both smooth and rough, with each showing different values.

For the purpose of researching the negative environmental impact of aircraft accidents up to 2000 kg MTOM, we focused on the evaluation of the composite material samples shown in Figure 1 and Table 1, in terms of the total smoke release (TSR) (m^2^/m^2^) and the total oxygen consumed (TOC) (g). Selected parameters, among all measured parameters, best describe the impact of a given material on air quality when burning. Based on the given parameters, which of the investigated composite materials in the combustion pollutes the air to the greatest extent was determined.

As already mentioned, this study focused on aircraft up to 2000 kg MTOM registered in the Czech Republic, namely: European Light Aircraft 1 (ELA1), European Light Aircraft 2 (ELA2), Light-Sport Aircraft (LSA), helicopters, gliders, motor gliders and ultra-light aircraft (ULL). Gliders were excluded from the group because of the minimal risk of fire during an emergency. A total of 103 aircraft types were investigated, including the main structural material of the aircraft. Based on the above, the percent of the aircraft surveyed using composite materials as the main construction material was evaluated.

## 3. Results

In the first phase of the research, data were collected on aircraft construction materials up to 2000 kg MTOM registered in the Czech Republic. Based on this, 103 different types of aircraft, helicopters, motor gliders, and ultra-light aircraft were analyzed. Of the analyzed types, the most typical types were ultra-light aircraft and light aircraft.

**Figure 2:**
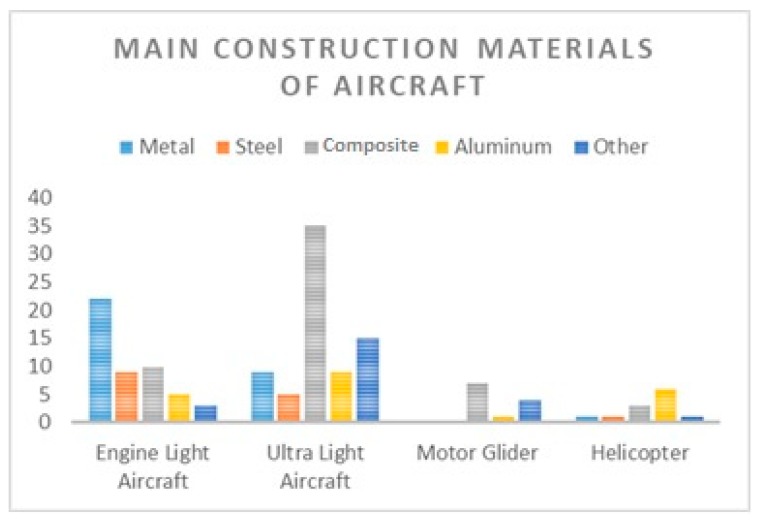
Construction materials of aircraft up to 2000 kg maximum take-off mass (MTOM).

Figure 2 shows the evaluation of the main construction material for the types under investigation by the register. The figure describes the representation of all major materials occurring in a particular type. If the airplane consisted of several major structural materials, all these materials were included in the list.

The structural composition of the analyzed aircraft is quite diverse. Within the engine light aircraft, metal prevails as the main structural material, or the structure is composed of canvas-coated steel tubes in combination with other materials. Most often, composite materials occur in wings, as all-composite wings provide less weight for an aircraft fuselage, thus improving flight performance and flight economy [32]. In helicopters registered in the Czech Republic, aluminum dominated as the main construction material, and it was mostly combined with some kind of composite material. In the structures of ultra-light aircraft and gliders, composites are the dominant material, especially fiberglass, which is combined with other lightweight materials.

As a result, we can see that composite materials are most commonly used in ultra-light aircraft structures, which is unambiguous with respect to their basic properties and parameters, especially in terms of weight, but their use is not negligible also in other types of aircraft. In aircraft and helicopter designs, composite materials appear in a smaller percentage even though they belong to the main structural material. Composite materials of different compositions, especially fiberglass and carbon composite, can be found in the types examined. Core and non-core composites are included, depending on the particular place of their use on the airplane.

Composite materials represent approximately 38% of all found main material designs of the investigated types of airplanes. Pure composite airplanes are found mainly in the group of motor gliders and ultra-light aircraft, where they represent approximately 49% of these groups. The remaining 51% are airplanes with a major construction material that combines a composite material with another material such as wood, metal, and aluminum.

On the basis of the above-mentioned facts and based on the fact that composite materials are increasingly involved in aircraft structures, their environmental impact needs to be assessed, particularly in the event of a fire in an air emergency.

The flammability tests performed on composite materials commonly used in aircraft structures were focused on several parameters. For the purpose of this study, we evaluated two parameters that best describe the environmental impact of a given material, namely air quality.

**Figure 3:**
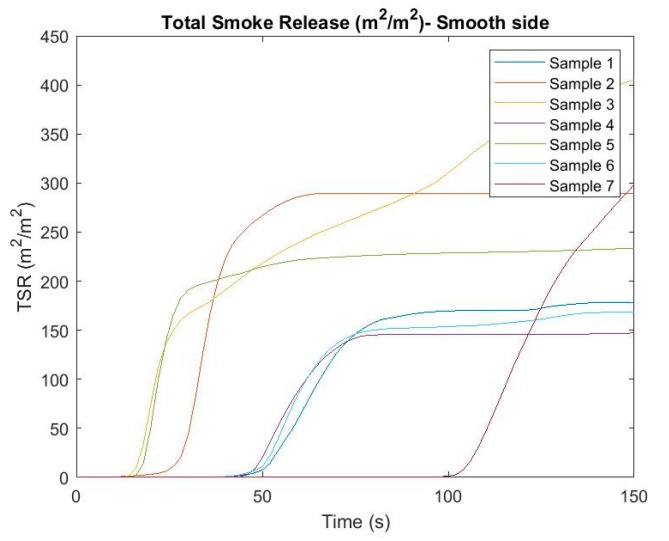
Total smoke release (m^2^/m^2^) in time—smooth side.

**Figure 4:**
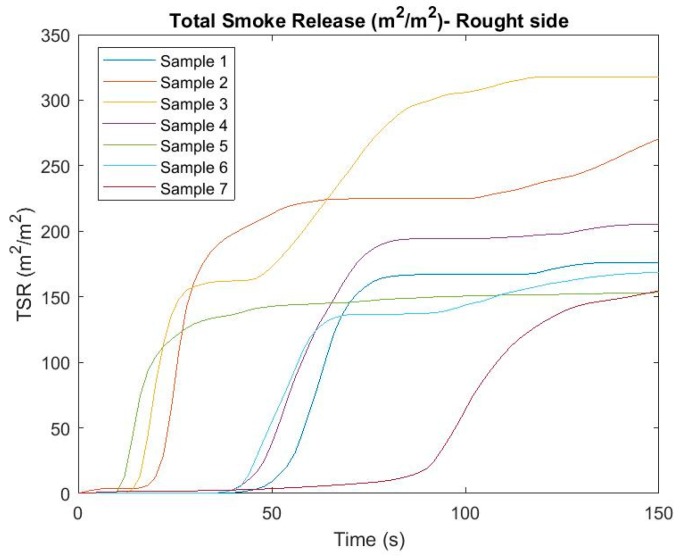
Total smoke release (m^2^/m^2^) in time—rough side.

In the flammability tests, one of the monitored and evaluated parameters was the amount of smoke released (m^2^/m^2^). Based on this parameter, we can see how much the air is contaminated when exposed to a particular material on fire. The parameter was evaluated for both sides of the heat (fire) radiation, while different values can be seen (Figure 3 and Figure 4). Figure 3 and Figure 4 describe the evolution of the amount of smoke released over time for individual materials and sides.

**Figure 5:**
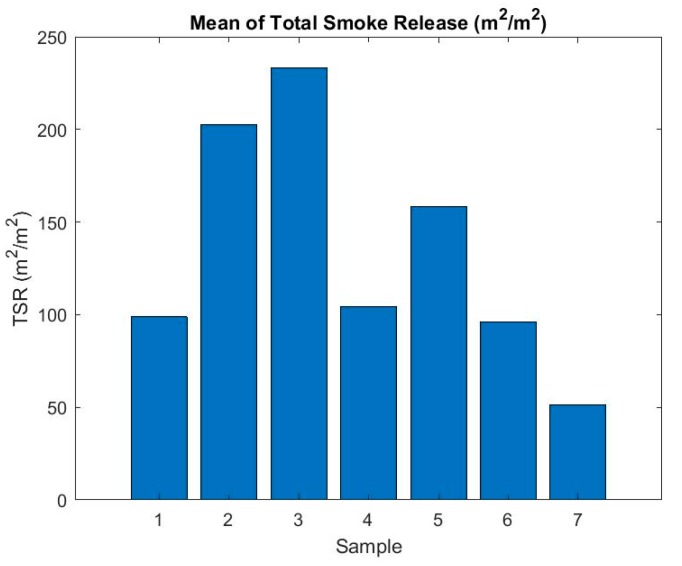
Mean of total smoke release (m^2^/m^2^).

Based on the figures above, we can see considerable differences in values based on the exposure side. Smoke was initiated from the rough side in most cases, rather than when the sample was exposed to fire radiation on a smooth side. Some of the samples produced an increasing volume of smoke, such as samples 2, 3, or 7, depending on the irradiation side. The volume of smoke in other samples, after reaching a certain time, mostly stagnated depending on the side of exposure. Obviously, due to the negative environmental impact of the material, the most susceptible samples are those with the longest fuming time and samples with increasing fume tendency over time. To better reflect the risk of each sample when evaluating a given parameter, the average TSR values for both sides were determined, thus achieving a comprehensive evaluation of the parameter for a particular sample. The results can be seen in Figure 5.

From the point of view of the TSR assessment over time, it can be seen that the riskiest composite material is sample number 3, which reached the highest value of the parameter during testing.

**Figure 6:**
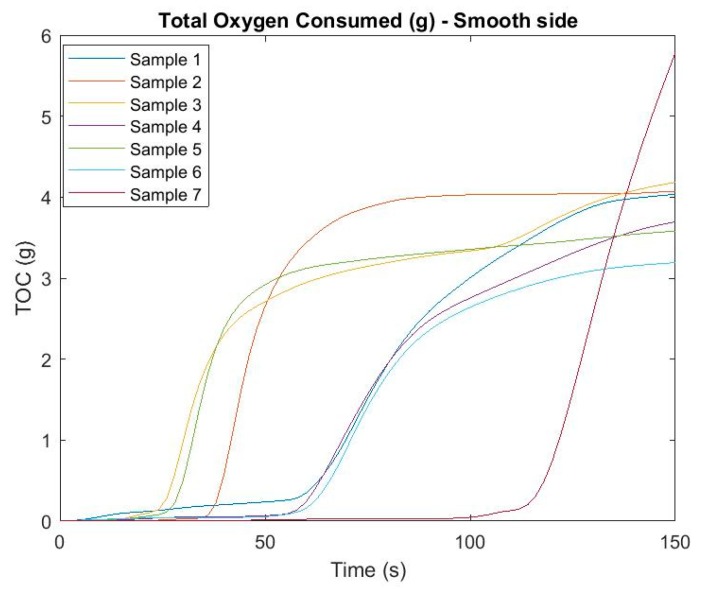
Total oxygen consumed (g) in time—smooth side.

**Figure 7:**
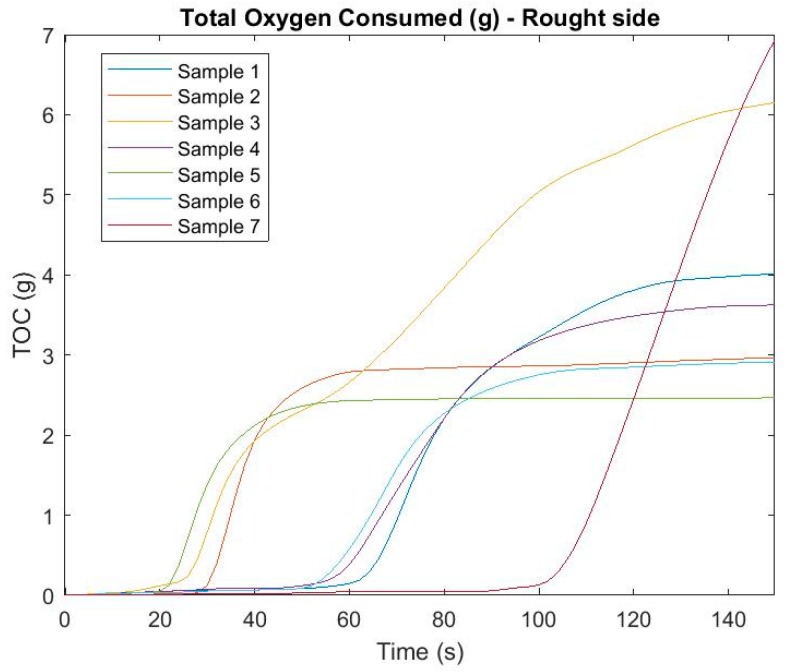
Total oxygen consumed (g) in time—rough side.

Another monitored parameter affecting air quality at the site of an emergency incident with fire is the amount of oxygen consumed (g) at the fire site. Again, samples from both sides were monitored, with Figure 6 and Figure 7 pointing to the development of TOC over time.

In this case, it is again possible to see differences at the time of initiation based on the side which was exposed to heat (fire) radiation. It was not proven that samples with an earlier initiation time, and therefore with a total longer burn time, consumed more oxygen for the duration of the measurement. It is best to see this in sample number 7, which started to consume oxygen up to approximately 100 s after exposure to the fire radiation, but ultimately consumed the most grams of oxygen. On the other hand, sample 5 belonged to one of the first where the data was measurable but at the end showed relatively low TOC values. Differences can also be observed between the smooth and rough sides of the samples. The biggest difference from the point of view of heat radiation was shown in sample no. 3.

**Figure 8:**
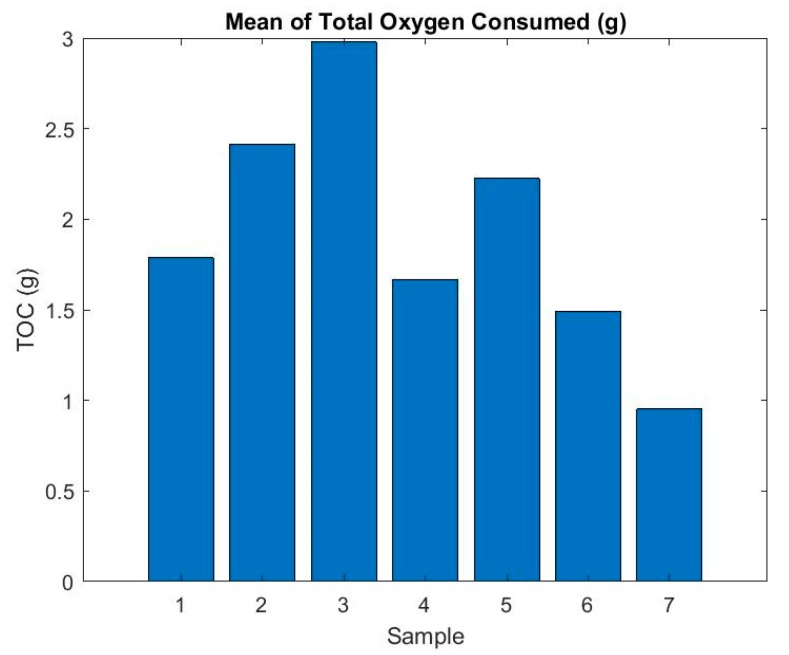
Mean of total oxygen consumed (g).

For a more accurate determination of the riskiest sample, TOC values for individual samples were again averaged based on which the sample could be evaluated comprehensively. It can be seen in Figure 8 that the riskiest sample is from the viewpoint of oxygen consumption—sample number 3, which consumed the largest volume of oxygen from the tested samples during testing.

Based on this, this study investigated the characteristics of composite materials in the combustion process and selected parameters to describe their environmental impact under fire conditions. Based on the research on the flammability and monitoring of selected parameters, we determined which components of the composite material in the burning process have the most negative impact on the environment. Within the monitored parameters, we focused on those that influence air pollution during combustion by releasing smoke and oxygen consumption. From the samples examined, sample number 3 was shown to be the riskiest material by observing both parameters. Sample 3 consists of 1× Carbon Fiber 43198 (198 g/m^2^) with Glass Fiber Surface (25 g/m^2^) + 8 mm Foam Core + 1× Carbon Fiber 43198 (198 g/m^2^), LG 385 epoxy resin binder + hardener HG 386, tempered. It is a composite material of a sandwich structure with a foam core.

## 4. Discussion

The fire resistance of materials is among the essential parameters that determine the inclusion of selected material in aircraft construction. Data on fire resistance of a particular material describes its properties under conditions of exposure to heat radiation or excessively high temperatures. Fire resistance is an important element that has a major impact on safety not only in a fire incident but in any event that causes a situation in which it is over-heated.

Currently, the most frequently used materials in aviation technology are composite materials of different composition (structure) with core and without core, depending on the place of their use. Many publications and research describe the basic properties of these materials. The question is, how the burning process of composite materials affects the environment, as most research has focused on improving the properties and resistance of composite materials under fire conditions. Smoke is produced while the material is burning and oxygen is lost at the place of fire, which has a major impact on air quality. The present research deals with the impact of commonly used composite materials on the environment, especially air quality during combustion. In the research, sample number 3 was defined as the most hazardous material, connecting Carbon Fiber with Glass Fiber Surface to a foam core. From the point of view of oxygen depletion at the place of combustion and smoke production, the sample showed the least favorable values. When comparing composite materials with and without core, logically, materials without a core exhibited better properties when evaluating both parameters. The values of composite materials with the core depend mainly on the type of core chosen.

It would be appropriate to build on this by expanding the research to determine what percentage of composite materials are in specific types of aircraft. In the analysis of the material composition of the designs of the aircraft types examined, this figure was not available for any aircraft. The final impact of a fire incident, in terms of air pollution, is based on the percentage of specific materials in the aircraft structure. However, since the main structural material of the aircraft was investigated, it is evident that the material in question has a major presence in the design of a particular aircraft. This research has shown that the composition of the composite material shown in Sample No. 3 is at the highest risk of impact on air quality and therefore the proportion of this material in the airplane structure should be reduced to the minimum possible level, possibly replaced by other less harmful material. Part of the supplementary research was also the analysis of air accidents, through which it was proven that in air accidents of aircraft up to 2000 kg MTOM in the Czech Republic, fires occur in only 12% of cases. However, on the basis of the above-mentioned facts, it is not possible to determine the future trend of fire incidents in aviation incidents, and that is why it is necessary to ensure the inclusion of more environmentally-friendly types of materials.

## 5. Conclusions

Safety and security are some of the most important aspects that concern transport [33,34]. Nowadays, with the increasing awareness of the negative impacts of the population on the environment, which is associated with global warming, ecology also comes to the fore. Many corporations place high demands on greening production, and in the transport environment, environmentally-friendly means or types of drives are being developed. One of the major negative environmental influences is air transport, the volume of which is constantly increasing. The enormous increase in traffic needs to be offset by the decreasing environmental impact of the used resources. That is why strict environmental standards are included in air transport. According to these facts, aviation manufacturers are looking for new technologies, materials, and drives that both reduce the negative environmental impact of aviation but are economically and operationally beneficial. From an economic and operational point of view, the best materials are composite materials that are characterized by low weight and good mechanical properties. Composite materials are currently one of the most widely used materials in the aerospace industry [35]. That is the reason that many studies deal with their inclusion in aircraft structures and their basic mechanical and thermal properties.

The present study analyzed the environmental impact of the combustion of selected composite materials. Seven samples of composite materials commonly used in aircraft designs up to 2000 kg MTOM were selected and tested on the basis of ISO 5660 using a cone calorimeter. Samples were selected based on the analysis of major aircraft construction materials up to 2000 kg MTOM registered in the Czech Republic. From the point of view of the influence of the given materials on the air quality at the place of burning of the given materials, two parameters were monitored and evaluated, namely the amount of smoke released (TSR) and the amount of oxygen consumed (TOC). On the basis of the flammability tests, it was found that sample no. 3, the composition of which combines carbon and fiberglass composite with a foam core, was least favorable for both parameters. The aim of the research was to determine the negative impact of selected types of composite materials on the air in the event of fire incidents.

On the basis of the results, it should be noted that it would be appropriate to maintain a database of accidents and incidents of general aviation aircraft that are more likely to occur in the future and analyze their impact on the environment as carried out in the case of commercial aircraft. It is evident that the extraordinary events and incidents of these aircraft have a negative impact not only on the air, but significantly damage the environment at the place of the event. Based on the research, the increasing use of composite materials has been outlined. As stated, flammability tests of composite materials used in aircraft structures (or other types of materials) are currently not being carried out, in particular with an assessment of their negative environmental impact during combustion. Based on the introduction of such material testing, those with the lowest environmental impact would be assessed as being dominant for aircraft structures. As part of further research, it is necessary to focus on the percentage evaluation of the use of specific composite materials in the structures of aircraft in service and, on this basis, to evaluate their negative impact on the air during combustion in the case of an aircraft incident/accident. Based on this, it is possible to design the composition of the aircraft composite structure so as to combine the positive ecological parameter of the composite materials.

## Figures and Tables

**Table 1:** List of samples.

Sample (Without Core)	Composition	Sample (With Core)	Composition
**1**	1× Fiberglass 92 110 (163 g/m^2^) + 2× Fiberglass 92 125 (285 g/m^2^), LG 385 epoxy resin binder + hardener HG 386, tempered.	**2**	1× Fiberglass 92 110 (163 g/m^2^) + 1× Fiberglass 92 125 (285 g/m^2^) + 8mm Foam Core + 1× Fiberglass 92 125 (285 g/m^2^), LG 385 epoxy resin binder + hardener HG 386, tempered.
**4**	3× Carbon Fiber (200 g/m^2^), LG 385 epoxy resin binder + hardener HG 386, tempered.	**3**	1× Carbon Fiber 43 198 (198 g/m^2^) with Glass Fiber surface (25 g/m^2^) + 8 mm Foam Core + 1× Carbon Fiber 43 198 (198 g/m^2^), LG 385 epoxy resin binder + hardener HG 386, tempered.
**6**	3× Hybrid Fabric CA 175 (Kevlar/carbon, 175 g/m^2^), LG 385 epoxy resin binder + hardener HG 386, tempered.	**5**	1× Fiberglass 92 110 (163 g/m^2^) + 1× Carbon Fiber (90 g/m^2^) + 8 mm Foam Core + 1× Carbon Fiber (90 g/m^2^), LG 385 Epoxy Resin Binder + Hardener HG 386, tempered.
		**7**	2× Hybrid Fabric CA 175 (Kevlar/carbon, 175 g/m^2^) + 1× Carbon Fiber (200 g/m^2^) + 4 mm Plywood Core + 1× Hybrid Fabric CA 175 (Kevlar/carbon, 175 g /m^2^) binder LG 385 epoxy resin binder + hardener HG 386, tempered.

## References

[1] EASA, EEA, EUROCONTROL (2016). European Aviation Environmental Report 2016.

[2] Jakubiak M. (2015). Environmental impact of air transport—Case study of Krakow Airport. Logistyka.

[3] Coffin A.W. (2007). From roadkill to road ecology: A review of the ecological effects of roads. J. Transp. Geogr..

[4] Macias A., Gadziński J. (2013). Assessment of Road Transport Environmental Impact as Illustrated by a Metropolitan Area. Pol. J. Environ. Stud..

[5] Sun Y., Hrušovský M., Zhang C., Lang M. (2018). A time-dependent fuzzy programming approach for the green multimodal routing problem with rail service capacity uncertainty and road traffic congestion. Complexity.

[6] Demir E., Burgholzer W., Hrušovský M., Arıkan E., Jammernegg W., Van Woensel T. (2016). A green intermodal service network design problem with travel time uncertainty. Transp. Res. Part B Methodol..

[7] Forsyth P. (2011). Environmental and financial sustainability of air transport: Are they incompatible?. J. Air Transp. Manag..

[8] Polishchuk V., Kelemen M., Gavurová B., Varotsos C., Andoga R., Gera M., Christodoulakis J., Soušek R., Kozuba J., Hospodka J. (2019). A fuzzy model of risk assessment for environmental start-up projects in the air transport sector. Int. J. Environ. Res. Public Health.

[9] Szabo S., Vittek P., Kraus J., Plos V., Lališ A., Štumper M., Vajdova I. (2017). Probabilistic model for airport runway safety areas. Transp. Probl..

[10] Koblen I., Szabo S., Krnáčová K. (2013). Selected information on European Union research and development programmes and projects focused on reducing emissions from air transport. Naše More.

[11] Zhu Q., Pei J., Liu X., Zhou Z. (2019). Analyzing commercial aircraft fuel consumption during descent: A case study using an improved K-means clustering algorithm. J. Clean. Prod..

[12] Cao X., OuYang S., Liu D., Yang W. (2019). Spatiotemporal Patterns and Decomposition Analysis of CO_2_ Emissions from Transportation in the Pearl River Delta. Energies.

[13] Liu H., Tian H., Hao Y., Liu S., Liu X., Zhu C., Wu Y., Liu W., Bai X., Wu B. (2019). Atmospheric emission inventory of multiple pollutants from civil aviation in China: Temporal trend, spatial distribution characteristics and emission features analysis. Sci. Total Environ..

[14] Matveev S.S. (2019). Simulation of CO and CO_2_ emissions in model combustion chamber based on the combination LES and Reactor Network model. REEE 2018: 2018 International Conference on Renewable Energy and Environment Engineering.

[15] Dzunda M., Cekanova D., Cobirka L., Zak P., Dzurovcin P. Ecological aspects associated with an operation of aviation electronic support systems. Proceedings of the 2017 TRANSNAV 2017: Safety of Sea Transportation—Proceedings of the International Conference on Marine Navigation and Safety of Sea Transportation.

[16] Li Y. (2019). Analysis and Forecast of Global Civil Aviation Accidents for the Period 1942–2016. Math. Probl. Eng..

[17] Dutton S., Kelly D., Baker A. (2004). Composite Materials for Aircraft Structures.

[18] Dodds N., Gibson A.G., Dewhurst D., Davies J.M. (2000). Fire behaviour of composite laminates. Compos. Part A Appl. Sci. Manuf..

[19] Lyon R.E., Balaguru P.N., Foden A., Sorathia U., Davidovits J., Davidovics M. (1997). Fire-resistant aluminosilicate composites. Fire Mater..

[20] Mouritz P.A., Gibson G.A. (2006). Fire Properties of Polymer Composite Materials.

[21] Brown E.J., Braun E., Twilley W.H. (1988). Cone Calorimeter Evaluation of the Flammability of Composite Materials.

[22] Kozłowski R., Władyka-Przybylak M. (2008). Flammability and fire resistance of composites reinforced by natural fibers. Polym. Fort Adv. Technol..

[23] Chapple S., Anandjiwala R. (2010). Flammability of Natural Fiber-reinforced Composites and Strategies for Fire Retardancy: A Review. J. Thermoplast. Compos. Mater..

[24] Zhou X., Qiu S., Cai W., Liu L., Hou Y., Wang W., Song L., Wang X., Hu Y. (2019). Construction of hierarchical MoS2@TiO2 structure for the high performance bimaleimide system with excellent fire safety and mechanical properties. Chem. Eng. J..

[25] George P., Bhowmik S., Abraham M., Sriram P.K., Pitchan M.K., Ajeesh G. (2016). High-performance fire-resistant polymeric nanocomposite for aerospace applications. Proc. Inst. Mech. Eng. Part L J. Mater. Des. Appl..

[26] ICAO (2019). State of Global Aviation Safety. https://www.icao.int/safety/Documents/ICAO_SR_2019_final_web.pdf.

[27] International Organization for Standardization ISO 5660-1:2002 (2015). Reaction To Fire Tests—Heat Release, Smoke Production And Mass Loss Rate—Part 1: Heat Release Rate (Cone Calorimeter Method) And Smoke Production Rate (Dynamic Measurement).

[28] Cone Calorimeter (ISO 5660 ASTM E 1354) The Most Comprehensive Bench Scale Fire Test. http://www.tech-quality.com/images/pdf/FTT/cone.pdf.

[29] Lindholm J., Brink A., Hupa M. (2009). Cone Calorimeter—A Tool For Measuring Heat Release Rate. http://www.ffrc.fi/FlameDays_2009/4B/LindholmPaper.pdf.

[30] Twilley W.H., Babrauskas V. (2001). User’s Guide for the Cone Calorimeter.

[31] Tobisová A., Mikula B., Szabo S., Blaško D., Vajdová I., Jenčová E., Melníková L., Szabo S., Němec V. The Simulation of Fire and Rescue Services Operations by Airplane Accidents. Proceedings of the NTAD 2018—13th International Scientific Conference—New Trends in Aviation Development Proceedings.

[32] Semrád K., Lipovský P., Čerňan J., Jurčovič M. Analysis of all-composite wing design containing magnetic microwires. Proceedings of the MMaMS: 6th Conference on Modelling of Mechanical and Mechatronic Systems.

[33] Kelemen M., Szabo S., Vajdová I. Security Management in the Air Transport: Example of an Interdisciplinary Investigation of Special Security Questions. Proceedings of the CNDCGS 2018, International Scientific Conference, Challenges to National Defence in Contemporary Geopolitical Situation.

[34] Szabo S., Vittek P., Lališ A., Červená V. Aviation Safety Investment Assessment Utilizing Return on Investment and Bayesian Networks. Proceedings of the Central European Conference on Finance and Economics CEFE2015.

[35] Šmelko M., Spodniak M., Semrád K., Tulipán P., Lipovský P., Moucha V. Aeronautical Composite Construction Monitoring by Magnetic Microwires. Proceedings of the NTAD 2018: XIII International Scientific Conference—New Trends in Aviation Development.

